# Thiamine deficiency‐related neuropathy: A reversible entity from an endemic area

**DOI:** 10.1111/ene.16155

**Published:** 2023-11-29

**Authors:** Sobia Nisar, Irfan Yousuf wani, Umair Altaf, Umar Muzaffer, Ozaifa Kareem, Masood Tanvir, Mohd. Ashraf Ganie

**Affiliations:** ^1^ Department of Medicine Government Medical College and Associated SMHS Hospital Srinagar Srinagar India; ^2^ Department of Medicine Government Medical College Srinagar India; ^3^ Department of Pharmaceutical Sciences University of Kashmir Srinagar India; ^4^ Department of Endocrinology Sher‐i‐Kashmir Institute of Medical Sciences Srinagar India

**Keywords:** clinical response, lower limb weakness, thiamine deficiency, neuropathy

## Abstract

**Background and purpose:**

Despite thiamine deficiency being a lesser‐known entity in modern times, beriberi in various forms, including thiamine deficiency‐related neuropathy, remains endemic in Kashmir due to the consumption of polished rice as a staple food. This observational study investigates cases of peripheral neuropathy of unknown etiology and their potential responsiveness to thiamine administration.

**Methods:**

This prospective study enrolled adult patients presenting to the emergency department with weakness consistent with thiamine deficiency‐related neuropathy and conducted a therapeutic challenge with thiamine on 41 patients. Response to thiamine therapy was monitored based on subjective and objective improvements in weakness and power. Patients were divided into thiamine responders (*n* = 25) and nonresponders (*n* = 16) based on their response to thiamine therapy and nerve conduction studies.

**Results:**

Most of the baseline characteristics were similar between responders and nonresponders, except the responders exhibited lower thiamine levels and higher partial pressure of oxygen and lactate levels compared to nonresponders. All patients had a history of consuming polished rice and traditional salt tea. Although weakness in the lower limbs was present in both groups, nonresponders were more likely to exhibit weakness in all four limbs. Clinical improvement was observed within 24 h, but proximal muscle weakness persisted for an extended period of time.

**Conclusions:**

Thiamine deficiency‐related neuropathy presents with predominant lower limb weakness, exacerbated by vomiting, poor food intake, psychiatric illness, and pregnancy. Thiamine challenge should be followed by observation of clinical and biochemical response.

## Graphical abstract

A comprehensive graphical abstract illustrating thiamine deficiency related neuropathy.

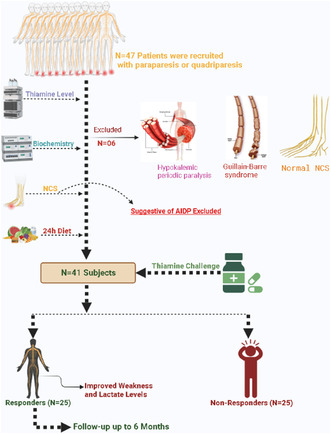

## INTRODUCTION

Beriberi is classified as dry and wet beriberi based on the neurological or cardiovascular manifestation [1]. Dry beriberi presents as Wernicke encephalopathy or thiamine deficiency‐related neuropathy (TDRN). Symptoms normally appear gradually over weeks to months, although they can sometimes appear suddenly over a few days, mimicking Guillain–Barré syndrome (GBS) [2]. Previous literature suggests that Asians are more likely to develop wet beriberi than dry beriberi [3], but the number of reported cases of both wet beriberi and dry beriberi with polyneuropathy has increased significantly in recent years among the Asian population. Thiamine deficiency is a well‐recognized public health issue in many communities in Southeast Asia (e.g., Cambodia, Laos, and Myanmar), South Asia (e.g., Nepal and Northern India), and West Africa [4, 5, 6, 7].

During the past decade, thiamine deficiency‐related disorders have been increasingly reported in the Kashmir valley in the northern part of India. These reports include case series of infantile beriberi [7] and polyneuropathy in peripartum women [8]. Previously, we have reported that High‐output heart failure patients responded to thiamine challenge with prompt resolution of metabolic abnormalities and clinical features. Although the manifestations of thiamine deficiency are varied and underreported, recently we have reported gastric beriberi as a new condition of thiamine‐responsive gastrointestinal upset [9]. Coupled with these reports, our observation of several cases of peripheral neuropathy of unknown etiology and responsiveness to thiamine administration prompted us to undertake this observational study.

## METHODOLOGY

This prospective study was conducted at a tertiary care hospital in Northern India, in accordance with the Helsinki Declaration of 1975. The institutional ethics committee approved the study (No.: 138/ETH/GMC/ICM, dated 28 May 2019), and informed consent was obtained from all participants. We have recruited adult patients (aged > 18 years) with weakness in the form of paraparesis or quadriparesis who presented to the emergency department from March 2020 to December 2022. Blood samples for thiamine level and biochemistry were taken followed by a thiamine challenge. A total of 47 patients were initially enrolled in the study, of whom six were excluded due to normal nerve conduction studies (NCS; *n* = 3), typical GBS (*n* = 1), or a past history suggestive of hypokalemic periodic paralysis (*n* = 2). The criteria utilized for determining normal and abnormal results in the context of NCS adhered to the guidelines outlined by Chen et al. [10]. Demyelinating neuropathy was distinguished by the hallmark features of diminished conduction velocity along with elongated distal and F‐wave latencies. In contrast, axonal neuropathy was typified by a decline in amplitude.

Therapeutic thiamine challenge, 600 mg by intravenous infusion, was given in normal saline every 8 h to the remaining 41 patients after obtaining samples for blood thiamine levels and biochemical parameters, and the response to thiamine was monitored at 24 h. NCS were performed within 24–48 h of admission. Based on the response to thiamine therapy and NCS (those suggestive of acute inflammatory demyelinating polyneuropathy were excluded), the patients were divided into two groups: thiamine responders (*n* = 25) and nonresponders (*n* = 16). Nonresponders were referred for further evaluation. Response was defined as subjective improvement in weakness and objective improvement in power and was monitored on a daily basis until the patient was discharged and followed on an outpatient department basis for 6 months. Details on demographics, clinical presentations, and history were obtained using a predesigned and validated study tool.

A qualified and trained dietician undertook a detailed diet review using a 24‐h recall to quantify various dietary components using specially designed diet software (Diet Cal, Profound Tech Solutions, New Delhi, India). The physician reviewed clinical assessment (anthropometry, general physical examination) and detailed neurological examination.

Blood samples at baseline were obtained for investigations such as complete blood count (XN‐1000 Haematology Analyser; Sysmex, Kobe, Japan), liver function test, kidney function test, electrolytes, and blood sugar samples, analysed using Architect *c*4000 (Abbott Laboratories, Abbott Park, IL). Lactate and venous blood gases assessment was made using fully automatic GEM Premier 3000 Blood Gas Analyzer (Werfen, Bedford, MA). Whole blood thiamine diphosphate/pyrophosphate levels were measured by high‐performance liquid chromatography using a fluorescence detector (Agilent‐1260; Agilent Technologies, Santa Clara, CA).

All NCS were conducted using a Nicolet EDX (Natus, Pleasanton, CA). The motor NCS employed low‐ and high‐frequency filter settings of 3 Hz and 10 kHz, whereas sensory NCS utilized filter settings of 20 Hz and 2 kHz. The temperature of the skin over the dorsum of the hand and foot was carefully maintained at a minimum of 30°C to ensure consistent conditions. Sensory NCS were carried out using an antidromic technique. For the radial nerve, the recording electrode was positioned along the nerve's trajectory through the anatomical snuffbox, with stimulating electrodes located 10 cm proximally along the radius. The sensory nerve action potentials of the median and ulnar nerves were captured using ring electrodes attached to digits II and V. Stimulation was applied at distances of 13 and 11 cm proximally from the wrist, respectively. Stimulation of the medial antebrachial cutaneous nerve occurred at the medial elbow, specifically between the medial epicondyle and the biceps tendon. The recording electrode was positioned 12 cm distally along the medial forearm. Similarly, the lateral antebrachial cutaneous nerve was stimulated within the antecubital fossa, with the recording electrode situated 12 cm distally along the lateral forearm. Posttreatment NCS were not done among all patients.

Statistical analysis was done using Statistical Package for Social Sciences version 26.0 (IBM, Armonk, NY). Normality was determined using Kolmogorov–Smirnov test. The mean and SD of the normally distributed variables are provided as mean ± SD, and nonnormal data are presented as the median and interquartile range. Paired *t*‐test was used to differentiate the lactate levels before and after the therapy. A *p*‐value ≤ 0.05 was taken as significant.

## RESULTS

The study recruited a total of 47 patients, six of whom were excluded based on normal NCS or typical GBS and a history suggestive of hypokalemic periodic paralysis. Based on their response to thiamine therapy, patients were classified into two groups, namely, responders (*n* = 25) and nonresponders (*n* = 16). Of the responders, nine were male and 16 were female, whereas among nonresponders, seven were male and nine were female. The average age of responders was 47.72 ± 17.33 years and of nonresponders was 46.81 ± 14.56 years. The baseline body weight of responders was 64.9 ± 6.9 kg and of nonresponders was 65.6 ± 11.2 kg. The mean baseline systolic (129.56 ± 28.25 and 131.00 ± 32.78 mmHg), diastolic (80.94 ± 21.17 and 82.25 ± 13.30 mmHg), and pulse pressure (86.44 ± 22.13 and 83.13 ± 14.86 mmHg) did not differ significantly between responders and nonresponders (Table 1).

**TABLE 1:** Baseline biochemical parameters of responder and nonresponder groups.

Parameter	Responders, *n* = 25, mean ± SD	Nonresponders, *n* = 16, mean ± SD	*p*
Age, years	47.72 ± 17.33	46.81 ± 14.56	0.870
SPO2%	95.42 ± 2.93	94.60 ± 4.51	0.664
Systolic blood pressure, mmHg	129.56 ± 28.25	131.00 ± 32.78	0.891
Diastolic blood pressure, mmHg	80.94 ± 21.17	82.25 ± 13.30	0.833
Pulse, bpm	86.44 ± 22.13	83.13 ± 14.86	0.616
Serum urea, mg/dL	50.57 ± 41.26	34.00 ± 14.51	0.155
Serum creatinine, mg/dL	1.60 ± 1.90	0.85 ± 0.35	0.146
Serum total bilirubin, mg/dL	1.12 ± 1.03	0.65 ± 0.31	0.177
Serum total protein, g/dL	7.02 ± 0.78	7.24 ± 0.99	0.577
Serum albumin, g/dL	3.46 ± 1.00	3.71 ± 0.59	0.508
Phosphate	3.30 ± 0.54	3.31 ± 1.31	0.987
Uric acid	3.77 ± 1.02	5.31 ± 1.43	0.127
SGOT (AST), IU/L	50.43 ± 52.29	38.50 ± 33.34	0.537
SGPT (ALT), IU/L	82.62 ± 121.67	31.10 ± 13.78	0.199
Serum ALP, IU/L	138.82 ± 80.66	89.20 ± 18.85	0.073
Serum sodium, mEq/L	137.29 ± 3.70	139.80 ± 3.85	0.085
Serum potassium, mEq/L	4.50 ± 0.69	3.06 ± 0.19	0.001
Blood glucose, random, mg/dL	120.62 ± 64.19	110.53 ± 72.54	0.702
Haemoglobin, g/dL	10.85 ± 2.86	12.00 ± 2.31	0.241
Neutrophil count, ×10^3^/μL	68.36 ± 12.41	73.22 ± 8.94	0.240
Red blood count, ×10^3^/μL	4.36 ± 1.63	4.57 ± 0.61	0.649
Lymphocyte count, %	25.10 ± 14.41	17.88 ± 7.58	0.100
White blood count, ×10^9^/L	9.19 ± 5.41	10.56 ± 5.51	0.506
SvO_2_, %	70.23 ± 23.15	63.27 ± 28.61	0.517
pH	7.32 ± 0.17	7.37 ± 0.11	0.413
pO_2_, mmHg	63.54 ± 9.14	41.45 ± 10.28	0.019
pCO_2_, mmHg	38.54 ± 7.18	33.91 ± 9.04	0.176
HCO_3_, mmol/L	21.00 ± 6.49	20.39 ± 6.70	0.821
Lactate, mmol/L	2.93 ± 0.37	1.99 ± 0.49	0.032
Blood thiamine diphosphate, μg/dL	0.99 ± 0.33	1.85 ± 0.38	0.015

Abbreviations: ALP, alkaline phosphatase; ALT, alanine aminotransferase; AST, aspartate aminotransferase; bpm, beats per minute; HCO_3_, bicarbonate; pCO_2_, partial pressure of carbon dioxide; pO_2_, partial pressure of oxygen; SGOT, serum glutamic–oxaloacetic transaminase; SGPT, serum glutamic pyruvic transaminase; SvO_2_, venous oxygen saturation .

Responders had significantly lower mean whole blood thiamine diphosphate levels (0.99 ± 0.33 μg/dL) compared to nonresponders (1.99 ± 0.38 μg/dL), although both groups had thiamine levels below normal. Thiamine responders had higher partial pressure of oxygen (pO_2_) (63.54% ± 9.14%) at presentation, which was statistically significant compared to nonresponders (41.45% ± 10.28%). The mean pH (7.32 ± 0.17 and 7.37 ± 0.11) and partial pressure of carbon dioxide (pCO_2_; 38.54 ± 7.18 and 33.91 ± 9.04) did not exhibit any significant difference between the responders and nonresponders. The biochemical parameters including urea, bilirubin, creatinine, and total protein were similar in both the responders and nonresponders. Nonresponders had lower venous oxygen saturation (63.27% ± 28.61%) than responders at presentation; this showed a statistically significant increase after administration of thiamine (71.31% ± 12.33%; Table 5). The mean lactate was significantly higher in responders (2.93 ± 0.37 mmol/L) than in nonresponders (1.99 ± 0.49 mmol/L), and the mean lactate drop following thiamine therapy was greater in responders (Table 5). The mean pH, pCO_2_, and bicarbonate were comparable between groups, whereas responders had elevated pO_2_ compared to nonresponders (Table 1) and the mean pO_2_ drop following thiamine therapy was statistically significantly greater among responders.

Vomiting, weakness, altered sensorium, and pedal oedema were more often reported as initial symptoms among responders than nonresponders. In contrast, abnormal sensation, difficulty maintaining balance, and decreased urine output were reported in both responders and nonresponders (Table 2).

**TABLE 2:** Baseline clinical characteristics of responder and nonresponder groups.

Characteristic	Nonresponders, *n* = 16, % (*n*)	Responders, *n* = 25, % (*n*)
Recurrent vomiting	18.7 (3)	40 (10)
History of poor intake	12.5 (2)	36 (9)
Sex		
Male	43.8 (7)	36 (9)
Female	56.2 (9)	64 (16)
Proton pump inhibitor	25 (4)	4 (1)
Diuretics	12.5 (2)	0
General weakness	100 (16)	100 (25)
Abnormal sensations	56.2 (9)	64 (16)
Difficulty maintaining balance	81.2 (13)	76 (19)
Altered sensorium	6.2 (1)	20 (5)
PND	18.75 (3)	8 (2)
Orthopnoea	18.75 (3)	8 (2)
Oedema	31.25 (5)	44 (11)
Decreased urine output	25 (4)	24 (6)

Abbreviation: PND, polyneuropathy disability.

In both groups of patients, weakness of the lower limbs was observed. NCS analysis indicated axonal neuropathy in 96% and mixed demyelinating axonal polyneuropathy in 4% of responders, whereas among nonresponders, axonal neuropathy was detected in 37.5% and mixed demyelinating axonal neuropathy in 62.5% of cases (Table 4). Responders predominantly presented with weakness of the lower limbs, whereas nonresponders exhibited weakness in all four limbs. Vomiting and fever were identified as the triggering factors among responders. Clinical improvement was observed within 24 h; however, proximal muscle weakness was slow to improve and persisted for several months (Table 4).

Within the scope of the present investigation, five patients displayed concurrent manifestations of wet beriberi, and an additional two individuals showcased simultaneous occurrences of Wernicke encephalopathy. Furthermore, a singular case was documented wherein rhabdomyolysis was observed. Among the subjects under scrutiny, four were contending with psychiatric disorders, whereas an equivalent number of cases were attributed to neuropathy with an underlying pregnancy‐related etiology. An admission history of inadequate dietary intake was ascertained in five patients. Notably, four patients received a diagnosis encompassing gastric beriberi, which was subsequently linked to a state of thiamine‐responsive neuropathy.

All the patients had a history of consuming polished rice as their main staple diet, washed once to three times before cooking. Among responders, the average calories derived from carbohydrates per day was 1815.14 ± 127.23 kcal; for nonresponders, it was 2016.15 ± 67.55 kcal. The total thiamine consumption per day for responders was 0.51 ± 0.10 mg and for nonresponders was 0.55 ± 0.20 mg. In addition, all patients had a history of drinking traditional salt tea daily (Table 3). No history of alcohol intake was reported among the responder and nonresponder groups.

**TABLE 3:** Diet analysis of responders and nonresponders.

	Responders, *n* = 25	Nonresponders, *n* = 16
Protein, g	50.21 ± 07.52	56.12 ± 11.26
Total fat, g	33.10 ± 16.28	32.73 ± 9.28
Carbohydrate, g	331.81 ± 43.32	344.15 ± 31.11
Energy, kcal	1815.14 ± 127.23	2016.15 ± 67.55
Thiamine, mg	0.51 ± 0.10	0.55 ± 0.20

*Note*: Data are presented as mean ± SD.

**TABLE 4:** Neurological examination of responders and nonresponders.

Power	Group	Initial			Follow‐up
		Weakness present, % (*n*)	Weakness absent, % (*n*)		Weakness present [persistent proximal muscle weakness], % (*n*)
Power upper limb left	Nonresponders	75 (12)	25 (4)		
	Responders	0	100 (25)		0
Upper limb right	Nonresponders	75 (12)	25 (4)		
	Responders	0	100 (25)		0
Lower limb left	Nonresponders	75(12)	25 (4)		
	Responders	44 (11)	56 (14)		28 (7)
Lower limb right	Nonresponders	75 (12)	25 (4)		
	Responders	72 (18)	28 (7)		40 (10)
Reflexes		Normal, % (*n*)	Hypoactive, % (*n*)	Absent, % (*n*)	Normal, % (*n*)
Upper limb left	Nonresponders	25 (4)	50 (8)	25 (4)	
	Responders	100 (25)	0	0	100 (25)
Upper limb right	Nonresponders	25 (4)	50 (8)	25 (4)	
	Responders	100 (25)	0	0	100 (25)
Lower limb left	Nonresponders	25 (4)	50 (8)	25 (4)	
	Responders	56 (14)	40 (10)	4 (1)	100 (25)
Lower limb right	Nonresponders	25 (4)	50 (8)	25 (4)	
	Responders	28 (7)	40 (10)	32 (8)	100 (25)
		Axonal neuropathy, % (*n*)	Demyelinating Axonal neuropathy, % (*n*)		
NCS	Nonresponders	37.5 (6)	62.5 (10)	–	
	Responders	96 (24)	04 (1)	–	

NCV values						
Nerve/site	Latency, ms		Amplitude, mV		Velocity, m/s	
	Responders, *n* = 25, mean ± SD	Nonresponders, *n* = 16, mean ± SD	Responders, *n* = 25, mean ± SD	Nonresponders, *n* = 16, mean ± SD	Responders, *n* = 25, mean ± SD	Nonresponders, *n* = 16, mean ± SD
R Median: APB						
Wrist	2.83 ± 0.98	3.64 ± 1.78	9.45 ± 4.5899	9.73 ± 5.13	48.29 ± 2.04	42.00 ± 1.50
Elbow	–	–	8.31 ± 4.9665	7.80 ± 5.37		
R Ulnar: ADM						
Wrist	2.44 ± 0.51	3.87 ± 1.15	8.78 ± 3.0016	8.25 ± 3.88	52.43 ± 1.88	50.63 ± 1.22
Elbow	7.19 ± 1.05	7.38 ± 1.66	7.60 ± 2.8164	7.33 ± 4.54		
R COMM Peroneal: EDB						
Ankle	4.36 ± 1.41	4.27 ± 1.20	1.94 ± 0.81	3.68 ± 1.89	44.00 ± 2.30	40.10 ± 2.41
Fib head	11.13 ± 1.77	10.83 ± 2.52	1.66 ± 0.70	2.95 ± 1.08		
L COMM Peroneal: EDB						
Ankle	4.96 ± 1.64	4.30 ± 0.97	1.24 ± 0.57	3.48 ± 1.15	47.20 ± 1.26	42.13 ± 2.06
Fib head	11.32 ± 1.81	11.54 ± 2.29	1.12 ± 0.52	2.71 ± 1.34		
R Tibial (knee): AH						
Ankle	4.27 ± 0.68	5.93 ± 2.67	8.08 ± 2.12	6.85 ± 2.41	42.67 ± 2.42	38.38 ± 1.12
Knee	12.33 ± 1.58	14.91 ± 4.19	8.00 ± 2.72	6.05 ± 2.48		
L Tibial (knee): AH						
Ankle	3.19 ± 0.61	5.86 ± 1.94	8.04 ± 4.2688	7.91 ± 4.75	41.00 ± 2.24	36.75 ± 2.13
Knee	12.71 ± 2.45	13.74 ± 6.44	6.08 ± 3.2936	5.60 ± 4.25		

F wave	Responders, mean ± SD	Nonresponders, mean ± SD	*p*
Median right: APB	31.12 ± 2.44	25.89 ± 1.86	0.020
Tibial (knee): AH left	53.75 ± 8.20	55.27 ± 10.75	0.321
Tibial (knee): AH right	54.57 ± 3.96	56.48 ± 12.36	0.075

Abbreviations: ADM, abductor digiti minimi; AH, abductor hallucis; APB, abductor pollicis brevis; COMM, common; EDB, extensor digitorum brevis; Fib, Fibular; L, left; NCS, nerve conduction studies; R, right.

**TABLE 5:** Pre‐ and posttreatment comparison between responders and nonresponders.

	Responders, *n* = 21			Nonresponders, *n* = 16		
	Pretreatment, mean ± SD	Posttreatment, mean ± SD	*p*	Pretreatment, mean ± SD	Posttreatment, mean ± SD	*p*
SVO_2_, %	70.23 ± 23.15	72.17 ± 6.12	0.163	63.27 ± 28.61	71.31 ± 12.33	0.085
pH	7.32 ± 0.17	7.41 ± 0.13	0.078	7.37 ± 0.11	7.43 ± 0.09	0.093
pO_2_, mmHg	63.54 ± 9.14	38.61 ± 4.88	0.011	41.45 ± 10.28	40.15 ± 6.83	0.703
pCO_2_, mmHg	38.54 ± 7.18	39.14 ± 6.31	0.341	33.91 ± 9.04	36.11 ± 9.73	0.613
HCO_3_, mmol/L	21.00 ± 6.49	23.12 ± 3.78	0.097	20.39 ± 6.70	22.17 ± 2.13	0.216
Lactate, mmol/L	2.93 ± 0.37	1.36 ± 0.61	0.041	1.99 ± 0.49	1.45 ± 0.07	0.147

Abbreviations: HCO_3_, bicarbonate; pCO_2_, partial pressure of carbon dioxide; pO_2_, partial pressure of oxygen; SVO_2_, mixed venous oxygen saturation.

## DISCUSSION

Beriberi with its various manifestations, including infantile beriberi, Wernicke encephalopathy [11], high‐output heart failure [12], gastric beriberi [9], and pregnancy‐related beriberi, is being reported in Kashmir valley, and the number of documented cases is increasing. TDRN, a subtype of dry beriberi being one of the manifestations, is also reported in the Kashmir valley [8], but has not been well described. There have been case reports of neuropathy associated with thiamine deficiency from various regions around the globe [2, 13], but an accurate description of the disease as a whole is not clearly defined. There is a possibility of missing thiamine‐related neuropathy due to conservatism in diagnosis or lack of well‐defined clinical criteria and unavailability of thiamine testing. We aimed to present the clinical profile of thiamine‐related neuropathy to facilitate diagnosis in the absence of thiamine testing.

In our study, the risk factors for TDRN were prolonged vomiting, loss of appetite, underlying psychiatric illness, and pregnancy. Recurrent and prolonged vomiting from any cause predisposes to thiamine deficiency. Among the patients presenting with TDRN, 40% had recurrent vomiting not responding to usual treatment. The patients were found to be thiamine deficient and responded well to thiamine challenge. Our study suggests vomiting as the commonest contributing factor of TDRN, which is in line with previous studies that reported vomiting as a forme fruste of beriberi [14, 15]. We have also recently reported gastric beriberi as a form of dry beriberi [9]. Nine patients among responders in our study had a history of poor intake of food for the past few days before presenting to hospital, due to either psychiatric illness or general loss of appetite. Psychiatric disease may be a predisposition due to poor intake. It has previously been reported that conditions that lead to poor food intake increase the risk of thiamine deficiency [16, 17, 18, 19]. Thus, improper and unbalanced nutrition for a few days to weeks or more may cause a severe form of thiamine deficiency [3]. Pregnancy is a known state of high demand for thiamine because of increasing metabolic needs of the fetus and the mother, and various forms of beriberi have been described in this state [20]. Six patients among TDRN in our study had pregnancy‐related neuropathy and presented with acute onset of lower limb weakness. This observation is in line with our previous studies [21, 22] and reports of thiamine deficiency in pregnant women of Karen [23], Gambia [5], and other communities [24].

In the current study, five patients were observed to have concomitant wet beriberi, and two exhibited co‐occurring Wernicke encephalopathy and one presented with rhabdomyolysis. The presence of concomitant wet beriberi has been previously documented by Prakasha et al. [25], who reported three cases with manifestations of right heart failure, shock, metabolic acidosis, and renal failure in varying combination with paraparesis, and successful recovery through prompt intervention with thiamine supplementation within a range of 24 h to several weeks. Furthermore, the co‐occurrence of Wernicke encephalopathy and neuropathy has also been reported in research conducted by Söylemez et al. [26], in a patient with a history of chronic daily alcohol consumption. Notably, in the current study, none of the patients exhibited a history of alcohol consumption.

The biochemical parameters of the patients presented in this study were characterized by lactate elevation (hyperlactataemia), resulting from the shift to anaerobic glycolysis [27, 28]. The mean lactate levels of the patients in the TDRN group were found to be higher than those of the nonresponders, which is a consistent phenomenon observed in previous research [9, 12, 29, 30]. Of particular interest was the significant drop of lactate in the responder group after thiamine challenge, which is consistent with findings from our previous studies in thiamine deficiency‐related high‐output heart failure [12] and gastric beriberi [9]. Furthermore, pO_2_ in venous blood was significantly higher (63.54 ± 9.14) among responders than nonresponders; these results corroborate the findings of our previous studies [9, 12].

The present observation found that the mean thiamine levels were lower in both TDRN and nonresponder groups, with TDRN patients showing the lowest thiamine levels and exhibiting a significant improvement after thiamine administration, suggesting neuropathy purely due to thiamine deficiency. However, absence of well‐defined cutoffs for thiamine levels could be the reason for the lower levels observed, highlighting the need for further studies to establish appropriate cutoff values. The nonresponder group had moderately higher levels of thiamine than the responder group and no response to thiamine administration, suggesting that lower thiamine levels in the absence of lactate elevation may not lead to metabolic derangement significant enough to cause neuropathy.

The predominant clinical manifestation in our study was weakness of the lower limbs with relative sparing of the upper limbs in the patients belonging to the thiamine‐responsive group. Previously, Hegde et al. [31] also observed predominant weakness of lower limbs and absence of sensory symptoms as the manifestation of thiamine deficiency. Sensory symptoms were absent in most of the cases, accompanied by notable upper limb sparing, which served as distinguishing factors from other acute and subacute neuropathies.

The NCS findings were characterized by presence of a significantly higher incidence of motor axonal neuropathy involving lower limb nerves among the TDRN group (96%) compared to nonresponders (37.5%), consistent with the findings of a previous study by Chen et al. [10].

Although myopathy has been previously documented in alcoholics, attributed to poor nutrition and muscle oedema, the role of thiamine deficiency has not been evaluated [32]. Our study lacks conclusive evidence attributing proximal muscle weakness to skeletal muscle impairment, as the levels of creatine phosphokinase (CPK) and lactate dehydrogenase (LDH) fell within the normal range. Nerve conduction velocity tests of the femoral nerve if performed could have provided further insights; however, we have documented instances of thiamine deficiency‐related rhabdomyolysis in other studies involving thiamine deficiency‐related disorders, although the extent of muscle damage in those cases exhibited elevations of LDH and CPK ranging from 10 to 46 times normal levels. In the present study, the clinical response to thiamine treatment was rapid, except for proximal muscle weakness showing a slow response that persisted for a period of 6 weeks to 6 months. The responder group had lower limb weakness with sparing of the upper limbs and most patients presented with paraparesis. Some cases were admitted with multimorbidity, including some with decompensating illness and TDRN observed as a co‐occurrence. The severity of weakness ranged from grade 0 power to grade 3 power, accompanied by areflexia or hyperreflexia.

All the patients had a history of consuming polished rice as their main staple diet, washed once to three times before cooking. Among responders, the average calories derived from carbohydrates per day was 1815.14 ± 127.23 kcal, whereas for nonresponders it was 2016.15 ± 67.55 kcal. The total thiamine consumption per day for responders was 0.51 ± 0.10 mg and for nonresponders was 0.55 ± 0.20 mg. The percent of calories derived from carbohydrates was low among responders as compared to nonresponders. In addition, all patients had a history of drinking traditional salt tea daily. No history of alcohol intake was reported among the responder and nonresponder groups.

## CONCLUSIONS

Based on the observations from this study, TDRN is a distinct manifestation of thiamine deficiency and can present with a range of symptoms, including lower limb weakness with sparing of the upper limbs. Prolonged vomiting, poor food intake, underlying psychiatric illness, and pregnancy are identified as key risk factors for TDRN. The biochemical parameters, such as elevated lactate levels and higher pO_2_ in venous blood, are suggestive of thiamine deficiency, or TDRN. Thiamine challenge is found to be an effective diagnostic and therapeutic tool for TDRN, with responders showing significant improvement in clinical and biochemical parameters. Overall, the findings from this study highlight the importance of early recognition and prompt management of thiamine deficiency to prevent the development of TDRN and associated complications.

### Recommendations and future directions

Addressing TDRN in regions like Kashmir requires a comprehensive approach. Public health initiatives should prioritize nutritional awareness, especially among polished rice‐dependent populations, emphasizing diversified diets and thiamine‐rich food fortification. Health care providers in endemic areas must implement routine thiamine deficiency screening, focusing on symptomatic individuals, using thiamine levels and biomarkers for early detection. Personalized treatment strategies considering individual characteristics are crucial. Long‐term follow‐up is essential for persistent symptoms. Future research should explore TDRN mechanisms for targeted interventions. Given its treatable nature, heightened suspicion in diagnosis is vital. In areas with thiamine deficiency prevalence, screening and fortification should be key preventive measures.

## FUNDING INFORMATION

This study is a part of the ICMR (Nutrition Division, Indian Council of Medical Research)‐funded study entitled, “Evaluation of Prevalence, Risk Factors, Consequences of Thiamine Deficiency Among Apparently Healthy Community‐Dwelling Kashmiri Population and Clinically Suspected Thiamine Deficiency Patients Attending Three Major Tertiary Care Hospitals: A Cross‐Sectional Study” (5/9/1206/2019‐Nut).

## CONFLICT OF INTEREST STATEMENT

We declare no conflicts of interest among authors related to this manuscript.

## Data Availability

The data that support the findings of this study are available from the corresponding author upon reasonable request.
